# Ammonium hydrogen (*RS*)-[(5-methyl-2-oxo-1,3-oxazolidin-3-yl)meth­yl]phospho­nate

**DOI:** 10.1107/S1600536809050338

**Published:** 2009-12-04

**Authors:** Petar Todorov, Emilia Naydenova, Rositsa P. Nikolova, Boris L. Shivachev

**Affiliations:** a University of Chemical Technology and Metallurgy, Department of Organic Chemistry, 8 Kl. Ohridski Boulevard, 1756 Sofia, Bulgaria; b Central Laboratory of Mineralogy and Crystallography, Bulgarian Academy of Sciences, Acad G. Bonchev Str. build. 107, 1113 Sofia, Bulgaria

## Abstract

In the title compound, NH_4_^+^·C_5_H_9_NO_5_P^−^, the five-membered methyl­oxazolidin-2-one unit is disordered over two positions, the major component having a site occupancy of 0.832 (9). A three-dimensional network of O—H⋯O and N—H⋯O hydrogen bonds stabilizes the crystal structure.

## Related literature

For general background of the use of phospho­nic and amino­phospho­nic acids as chelating agents in metal extraction and as medicinal compounds, see: Metlushka *et al.* (2009 ▶); Naydenova *et al.* (2009 ▶); Matczak-Jon & Videnova-Adrabinska (2005 ▶). For related structures, see: Dudko *et al.* (200 ▶9); Shivachev *et al.* (2005 ▶); Todorov *et al.* (2006 ▶); Ying *et al.* (2007 ▶). For bond-length data, see: Allen *et al.* (1987 ▶).
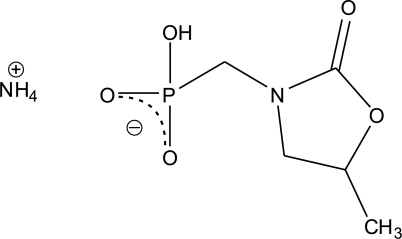


## Experimental

### 

#### Crystal data

NH_4_^+^·C_5_H_9_NO_5_P^−^
*M* *_r_* = 212.14
Triclinic, 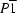

*a* = 6.471 (3) Å
*b* = 8.801 (3) Å
*c* = 9.427 (4) Å
α = 70.76 (2)°
β = 70.658 (18)°
γ = 89.363 (16)°
*V* = 475.4 (3) Å^3^
*Z* = 2
Mo *K*α radiation
μ = 0.29 mm^−1^
*T* = 290 K
0.30 × 0.28 × 0.21 mm

#### Data collection

Enraf–Nonius CAD-4 diffractometer
Absorption correction: none
3673 measured reflections
1855 independent reflections
1606 reflections with *I* > 2σ(*I*)
*R*_int_ = 0.027
3 standard reflections frequency: 120 min intensity decay: −1%

#### Refinement

*R*[*F* ^2^ > 2σ(*F* ^2^)] = 0.035
*wR*(*F* ^2^) = 0.097
*S* = 1.05
1855 reflections
159 parameters
H-atom parameters constrained
Δρ_max_ = 0.24 e Å^−3^
Δρ_min_ = −0.33 e Å^−3^

### 

Data collection: *CAD-4 EXPRESS* (Enraf–Nonius, 1994 ▶); cell refinement: *CAD-4 EXPRESS*; data reduction: *XCAD4* (Harms & Wocadlo, 1995 ▶); program(s) used to solve structure: *SHELXS97* (Sheldrick, 2008 ▶); program(s) used to refine structure: *SHELXL97* (Sheldrick, 2008 ▶); molecular graphics: *ORTEP-3 for Windows* (Farrugia, 1997 ▶) and *Mercury* (Macrae *et al.*, 2006 ▶); software used to prepare material for publication: *WinGX* (Farrugia, 1999 ▶).

## Supplementary Material

Crystal structure: contains datablocks I, global. DOI: 10.1107/S1600536809050338/is2491sup1.cif

Structure factors: contains datablocks I. DOI: 10.1107/S1600536809050338/is2491Isup2.hkl

Additional supplementary materials:  crystallographic information; 3D view; checkCIF report

## Figures and Tables

**Table 1:** Hydrogen-bond geometry (Å, °)

*D*—H⋯*A*	*D*—H	H⋯*A*	*D*⋯*A*	*D*—H⋯*A*
N2—H*N*1⋯O1	0.85	1.94	2.789 (2)	177
O2—H1*A*⋯O3^i^	1.07	1.53	2.5770 (19)	166
N2—H*N*2⋯O1^ii^	0.86	1.93	2.772 (2)	165
N2—H*N*3⋯O3^iii^	0.89	1.93	2.793 (2)	161
N2—H*N*4⋯O4^iv^	0.97	1.88	2.827 (2)	167

Symmetry codes: (i) 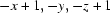; (ii) 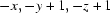; (iii) 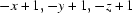; (iv) 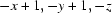.

**Fig. 1.**
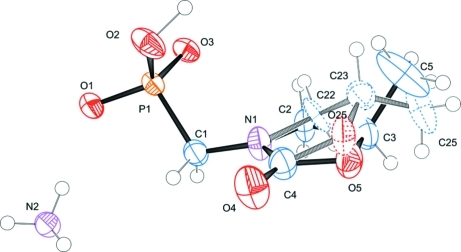
The asymmetric unit of title compound with the atom numbering scheme showing 50% probability displacement ellipsoids. H atoms are shown as small spheres of arbitrary radii. Minor occupancy disorder component is represented with dashed lines.

**Fig. 2.**
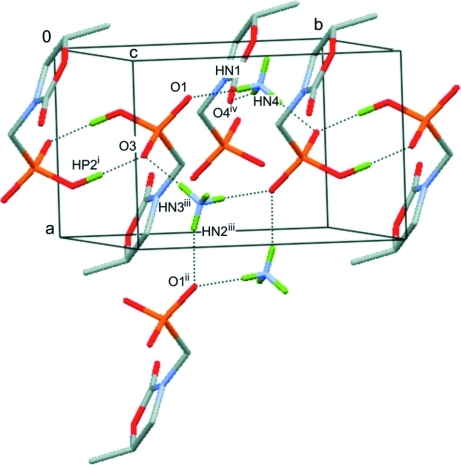
A view of the molecular packing in the title compound. All H atoms not involved in the short contact interactions have been omitted for clarity. [Symmetry codes: (i) -x + 1, -y, z + 1; (ii) 1 + x, -y + 1, -z + 1; (iii) -x + 1, -y + 1, -z + 1; (iv) -x + 1, -y + 1, -z.]

## supplementary crystallographic information

### Comment

Phosphonic and aminophosphonic derivatives have a high potential for biological activity. These derivatives have been widely used in the manufacture of herbicides, as chelating agents in metal extraction and as medicinal compounds (Metlushka *et al.*, 2009; Naydenova *et al.*, 2009; Matczak-Jon & Videnova-Adrabinska, 2005). As part of our ongoing studies of the structure-activity relationships for phosphonic acid derivatives (Todorov *et al.*, 2006; Shivachev *et al.*, 2005) herein we report the structure of the titled compound.

The asymmetric unit of the title compound (Fig. 1) contains one molecule, with a proton transferred from the phosphonic group to the ammonia group. The ammonium cation attendant in structure neutralizes the negatively charged phosphonic acid residue. In the crystal structure, the methyloxazolidin-2-one moiety is disordered over two positions. In one of them (major occupancy) the oxazolidine ring (N1/C2/C3/O5/c4) is almost planar [r.m.s. of 0.017 (2) Å] while in the other one it adopts an envelope conformation, with atom C22 deviating within 0.367 (33) Å from the plane defined by the other four atoms [N1/C4/O25/C23 with r.m.s. 0.006 (5) Å]. Bond lengths and angles have normal values and compare well with related structures (Allen *et al.*, 1987; Dudko *et al.*, 2009; Ying *et al.*, 2007; Todorov *et al.*, 2006). The phosphorus atom displays a slightly distorted tetrahedral geometry provided by three oxygen atoms and one carbon atom.

The crystal structure of title compound shows three-dimensional network of O—H···O and N—H···O hydrogen bonds which additionally stabilize the structure (Table 1 and Fig. 2).

### Experimental

The title compound, NH_4_^+^.C_5_H_10_N_2_O_5_P^-^, was obtained from the reaction of 5-methyloxazolidin-2-one with formaldehyde and phosphorus trichloride in glacial acetic acid. The solution was left at room temperature. Colorless crystals of the title compound were obtained after several days staying.

### Refinement

The hydroxy and ammonium H atoms were located in a difference map. H atoms bonded to C were placed in idealized positions (C—H = 0.97 Å for CH_3_, C—H = 0.96 Å for CH_2_ and C—H = 0.98 Å for CH). All H atoms were constrained to ride on their parent atoms, with *U*_iso_(H) = 1.2*U*_eq_(C, O) and *U*_iso_(H) = 1.5*U*_eq_(methyl C). The *U*_iso_(H) values of N-bound H atoms were freely refined.

### Crystal data

**Table:**

NH_4_^+^·C_5_H_9_NO_5_P^−^	*Z* = 2
*M**_r_* = 212.14	*F*(000) = 224
Triclinic, *P*1	*D*_x_ = 1.482 Mg m^−^^3^
Hall symbol: -P 1	Melting point: not measured K
*a* = 6.471 (3) Å	Mo *K*α radiation, λ = 0.71073 Å
*b* = 8.801 (3) Å	Cell parameters from 22 reflections
*c* = 9.427 (4) Å	θ = 18.2–19.9°
α = 70.76 (2)°	µ = 0.28 mm^−^^1^
β = 70.658 (18)°	*T* = 290 K
γ = 89.363 (16)°	Prismatic, pale yellow
*V* = 475.4 (3) Å^3^	0.30 × 0.28 × 0.21 mm

### Data collection

**Table:**

Enraf–Nonius CAD-4 diffractometer	*R*_int_ = 0.027
Radiation source: fine-focus sealed tube	θ_max_ = 26.0°, θ_min_ = 2.4°
graphite	*h* = −7→7
ω/2θ scans	*k* = −10→10
3673 measured reflections	*l* = −11→11
1855 independent reflections	3 standard reflections every 120 min
1606 reflections with *I* > 2σ(*I*)	intensity decay: −1%

### Refinement

**Table:**

Refinement on *F*^2^	Primary atom site location: structure-invariant direct methods
Least-squares matrix: full	Secondary atom site location: difference Fourier map
*R*[*F*^2^ > 2σ(*F*^2^)] = 0.035	Hydrogen site location: inferred from neighbouring sites
*wR*(*F*^2^) = 0.097	H-atom parameters constrained
*S* = 1.05	*w* = 1/[σ^2^(*F*_o_^2^) + (0.0517*P*)^2^ + 0.1432*P*] where *P* = (*F*_o_^2^ + 2*F*_c_^2^)/3
1855 reflections	(Δ/σ)_max_ = 0.001
159 parameters	Δρ_max_ = 0.24 e Å^−^^3^
0 restraints	Δρ_min_ = −0.33 e Å^−^^3^

### Special details

**Table:**

Geometry. All e.s.d.'s (except the e.s.d. in the dihedral angle between two l.s. planes) are estimated using the full covariance matrix. The cell e.s.d.'s are taken into account individually in the estimation of e.s.d.'s in distances, angles and torsion angles; correlations between e.s.d.'s in cell parameters are only used when they are defined by crystal symmetry. An approximate (isotropic) treatment of cell e.s.d.'s is used for estimating e.s.d.'s involving l.s. planes.
Refinement. Refinement of *F*^2^ against ALL reflections. The weighted *R*-factor *wR* and goodness of fit *S* are based on *F*^2^, conventional *R*-factors *R* are based on *F*, with *F* set to zero for negative *F*^2^. The threshold expression of *F*^2^ > σ(*F*^2^) is used only for calculating *R*-factors(gt) *etc*. and is not relevant to the choice of reflections for refinement. *R*-factors based on *F*^2^ are statistically about twice as large as those based on *F*, and *R*- factors based on ALL data will be even larger.

### Fractional atomic coordinates and isotropic or equivalent isotropic displacement parameters (Å^2^)

**Table:**

	*x*	*y*	*z*	*U*_iso_*/*U*_eq_	Occ. (<1)
P1	0.41857 (7)	0.24449 (5)	0.45738 (5)	0.02797 (16)	
O3	0.5389 (2)	0.15751 (15)	0.56719 (15)	0.0337 (3)	
O1	0.2459 (2)	0.34533 (16)	0.51681 (16)	0.0398 (3)	
O4	0.6905 (3)	0.3598 (2)	−0.03575 (18)	0.0551 (4)	
O2	0.3153 (2)	0.12049 (16)	0.40620 (19)	0.0451 (4)	
H1A	0.3957	0.0129	0.4192	0.054*	
N1	0.7900 (3)	0.3121 (2)	0.18552 (19)	0.0384 (4)	
C4	0.8154 (3)	0.3152 (3)	0.0385 (2)	0.0409 (5)	
C1	0.6134 (3)	0.3843 (2)	0.2730 (2)	0.0344 (4)	
H1B	0.5331	0.4384	0.2035	0.041*	
H1C	0.6778	0.4664	0.2985	0.041*	
N2	0.2060 (2)	0.66240 (18)	0.34358 (18)	0.0328 (4)	
HN2	0.0704	0.6790	0.3837	0.045 (6)*	
HN1	0.2222	0.5670	0.3972	0.052 (7)*	
HN3	0.3009	0.7318	0.3485	0.056 (7)*	
HN4	0.2541	0.6712	0.2322	0.054 (7)*	
C2	0.9747 (8)	0.2620 (6)	0.2347 (6)	0.0391 (10)	0.832 (9)
H2A	0.9323	0.1667	0.3309	0.047*	0.832 (9)
H2B	1.0433	0.3480	0.2527	0.047*	0.832 (9)
O5	1.0145 (8)	0.2687 (6)	−0.0269 (6)	0.0502 (9)	0.832 (9)
C5	1.1711 (12)	0.0514 (6)	0.1183 (8)	0.094 (2)	0.832 (9)
H5A	1.2641	0.0400	0.0200	0.141*	0.832 (9)
H5B	1.0342	−0.0159	0.1578	0.141*	0.832 (9)
H5C	1.2432	0.0191	0.1961	0.141*	0.832 (9)
C3	1.1272 (5)	0.2245 (4)	0.0887 (3)	0.0421 (10)	0.832 (9)
H3	1.2677	0.2932	0.0443	0.050*	0.832 (9)
C22	0.988 (5)	0.221 (3)	0.217 (3)	0.039 (5)	0.168 (9)
H22A	1.1119	0.2984	0.1918	0.047*	0.168 (9)
H22B	0.9459	0.1536	0.3292	0.047*	0.168 (9)
O25	0.957 (3)	0.209 (3)	−0.005 (3)	0.043 (4)	0.168 (9)
C25	1.273 (4)	0.128 (3)	0.054 (3)	0.070 (7)	0.168 (9)
H25A	1.3078	0.0833	−0.0305	0.105*	0.168 (9)
H25B	1.3286	0.0648	0.1352	0.105*	0.168 (9)
H25C	1.3404	0.2376	0.0112	0.105*	0.168 (9)
C23	1.047 (3)	0.125 (2)	0.1196 (16)	0.041 (5)	0.168 (9)
H23	0.9758	0.0133	0.1781	0.049*	0.168 (9)

### Atomic displacement parameters (Å^2^)

**Table:**

	*U*^11^	*U*^22^	*U*^33^	*U*^12^	*U*^13^	*U*^23^
P1	0.0290 (3)	0.0249 (2)	0.0322 (3)	0.00831 (17)	−0.01321 (19)	−0.01013 (19)
O3	0.0414 (7)	0.0289 (6)	0.0372 (7)	0.0081 (5)	−0.0215 (6)	−0.0115 (6)
O1	0.0367 (7)	0.0324 (7)	0.0418 (8)	0.0125 (6)	−0.0063 (6)	−0.0097 (6)
O4	0.0615 (10)	0.0769 (11)	0.0407 (8)	0.0307 (9)	−0.0294 (8)	−0.0266 (8)
O2	0.0516 (8)	0.0327 (7)	0.0715 (10)	0.0147 (6)	−0.0440 (8)	−0.0214 (7)
N1	0.0343 (8)	0.0544 (10)	0.0328 (8)	0.0181 (8)	−0.0147 (7)	−0.0202 (8)
C4	0.0418 (11)	0.0482 (12)	0.0313 (10)	0.0140 (9)	−0.0122 (9)	−0.0129 (9)
C1	0.0363 (10)	0.0335 (9)	0.0314 (9)	0.0108 (8)	−0.0100 (8)	−0.0107 (8)
N2	0.0338 (9)	0.0310 (8)	0.0338 (8)	0.0066 (6)	−0.0128 (7)	−0.0108 (7)
C2	0.0315 (15)	0.054 (3)	0.0355 (17)	0.0178 (18)	−0.0107 (12)	−0.0209 (18)
O5	0.046 (2)	0.065 (2)	0.0321 (13)	0.0220 (15)	−0.0070 (15)	−0.0147 (17)
C5	0.158 (7)	0.063 (3)	0.130 (5)	0.064 (3)	−0.111 (5)	−0.059 (3)
C3	0.0309 (14)	0.045 (2)	0.0543 (17)	0.0107 (13)	−0.0152 (12)	−0.0220 (14)
C22	0.059 (9)	0.041 (11)	0.044 (8)	0.037 (8)	−0.042 (7)	−0.037 (8)
O25	0.034 (9)	0.067 (12)	0.035 (8)	0.023 (7)	−0.013 (7)	−0.025 (9)
C25	0.066 (14)	0.087 (18)	0.104 (19)	0.049 (11)	−0.050 (13)	−0.073 (16)
C23	0.045 (8)	0.037 (9)	0.043 (7)	0.009 (7)	−0.015 (6)	−0.016 (6)

### Geometric parameters (Å, °)

**Table:**

P1—O1	1.4969 (14)	C2—C3	1.539 (5)
P1—O3	1.5041 (13)	C2—H2A	0.9700
P1—O2	1.5645 (14)	C2—H2B	0.9700
P1—C1	1.816 (2)	O5—C3	1.454 (6)
O4—C4	1.218 (2)	C5—C3	1.497 (6)
O2—H1A	1.0666	C5—H5A	0.9600
N1—C4	1.332 (3)	C5—H5B	0.9600
N1—C2	1.435 (6)	C5—H5C	0.9600
N1—C1	1.447 (2)	C3—H3	0.9800
N1—C22	1.56 (2)	C22—C23	1.41 (2)
C4—O5	1.355 (5)	C22—H22A	0.9700
C4—O25	1.36 (2)	C22—H22B	0.9700
C1—H1B	0.9700	O25—C23	1.46 (3)
C1—H1C	0.9700	C25—C23	1.39 (3)
N2—HN2	0.8645	C25—H25A	0.9600
N2—HN1	0.8517	C25—H25B	0.9600
N2—HN3	0.8934	C25—H25C	0.9600
N2—HN4	0.9676	C23—H23	0.9800
			
O1—P1—O3	117.20 (8)	H2A—C2—H2B	109.3
O1—P1—O2	109.29 (9)	C4—O5—C3	109.8 (4)
O3—P1—O2	109.68 (8)	C3—C5—H5A	109.5
O1—P1—C1	104.86 (8)	C3—C5—H5B	109.5
O3—P1—C1	109.63 (9)	H5A—C5—H5B	109.5
O2—P1—C1	105.49 (10)	C3—C5—H5C	109.4
P1—O2—H1A	112.0	H5A—C5—H5C	109.5
C4—N1—C2	114.0 (2)	H5B—C5—H5C	109.5
C4—N1—C1	122.16 (16)	O5—C3—C5	108.1 (3)
C2—N1—C1	122.7 (2)	O5—C3—C2	104.9 (3)
C4—N1—C22	101.9 (8)	C5—C3—C2	115.8 (4)
C1—N1—C22	135.9 (8)	O5—C3—H3	109.2
O4—C4—N1	127.90 (19)	C5—C3—H3	109.2
O4—C4—O5	122.2 (3)	C2—C3—H3	109.3
N1—C4—O5	109.8 (3)	C23—C22—N1	108.1 (16)
O4—C4—O25	116.8 (9)	C23—C22—H22A	110.5
N1—C4—O25	111.6 (10)	N1—C22—H22A	110.4
N1—C1—P1	115.39 (13)	C23—C22—H22B	109.8
N1—C1—H1B	108.4	N1—C22—H22B	109.7
P1—C1—H1B	108.4	H22A—C22—H22B	108.3
N1—C1—H1C	108.4	C4—O25—C23	112.2 (16)
P1—C1—H1C	108.4	C23—C25—H25A	109.5
H1B—C1—H1C	107.5	C23—C25—H25B	109.5
HN2—N2—HN1	108.1	H25A—C25—H25B	109.5
HN2—N2—HN3	112.5	C23—C25—H25C	109.4
HN1—N2—HN3	108.1	H25A—C25—H25C	109.5
HN2—N2—HN4	115.2	H25B—C25—H25C	109.5
HN1—N2—HN4	107.4	C25—C23—C22	112 (2)
HN3—N2—HN4	105.2	C25—C23—O25	110.4 (17)
N1—C2—C3	101.4 (3)	C22—C23—O25	100.2 (16)
N1—C2—H2A	111.6	C25—C23—H23	111.1
C3—C2—H2A	111.5	C22—C23—H23	111.5
N1—C2—H2B	111.5	O25—C23—H23	111.2
C3—C2—H2B	111.4		
			
C2—N1—C4—O4	−176.3 (3)	O4—C4—O5—C3	179.2 (2)
C1—N1—C4—O4	−8.3 (4)	N1—C4—O5—C3	2.6 (4)
C22—N1—C4—O4	172.4 (11)	O25—C4—O5—C3	−96 (3)
C2—N1—C4—O5	0.1 (3)	C4—O5—C3—C5	120.3 (5)
C1—N1—C4—O5	168.1 (3)	C4—O5—C3—C2	−3.9 (4)
C22—N1—C4—O5	−11.2 (11)	N1—C2—C3—O5	3.7 (4)
C2—N1—C4—O25	26.4 (9)	N1—C2—C3—C5	−115.5 (5)
C1—N1—C4—O25	−165.6 (9)	C4—N1—C22—C23	−25 (2)
C22—N1—C4—O25	15.1 (13)	C2—N1—C22—C23	−165 (6)
C4—N1—C1—P1	117.3 (2)	C1—N1—C22—C23	156.3 (11)
C2—N1—C1—P1	−75.7 (3)	O4—C4—O25—C23	−161.7 (9)
C22—N1—C1—P1	−63.7 (15)	N1—C4—O25—C23	−1.7 (15)
O1—P1—C1—N1	−175.71 (13)	O5—C4—O25—C23	89 (3)
O3—P1—C1—N1	57.66 (16)	N1—C22—C23—C25	140 (2)
O2—P1—C1—N1	−60.36 (15)	N1—C22—C23—O25	22 (2)
C4—N1—C2—C3	−2.4 (4)	C4—O25—C23—C25	−132 (2)
C1—N1—C2—C3	−170.3 (2)	C4—O25—C23—C22	−14 (2)
C22—N1—C2—C3	41 (4)		

### Hydrogen-bond geometry (Å, °)

**Table:**

*D*—H···*A*	*D*—H	H···*A*	*D*···*A*	*D*—H···*A*
N2—HN1···O1	0.85	1.94	2.789 (2)	177
O2—H1A···O3^i^	1.07	1.53	2.5770 (19)	166
N2—HN2···O1^ii^	0.86	1.93	2.772 (2)	165
N2—HN3···O3^iii^	0.89	1.93	2.793 (2)	161
N2—HN4···O4^iv^	0.97	1.88	2.827 (2)	167

Symmetry codes: (i) −*x*+1, −*y*, −*z*+1; (ii) −*x*, −*y*+1, −*z*+1; (iii) −*x*+1, −*y*+1, −*z*+1; (iv) −*x*+1, −*y*+1, −*z*.
